# Abstract Concepts, Social Interaction, and Beliefs

**DOI:** 10.3389/fpsyg.2022.919808

**Published:** 2022-06-29

**Authors:** Anna M. Borghi, Chiara Fini, Claudia Mazzuca

**Affiliations:** ^1^Laboratory BALLAB (Body Action Language LAB), Department of Dynamic and Clinical Psychology and Health Studies, Sapienza University of Rome, Rome, Italy; ^2^Institute of Cognitive Sciences and Technologies, Italian National Research Council, Rome, Italy

**Keywords:** abstract concepts, belief, abstract words, Theory of Mind, social interaction, concepts, metacognition, categorization

## Introduction

This paper focuses on abstract concepts like “fantasy,” “self-determination,” and belief. Proficiently mastering abstract concepts and the words expressing them is a complex ability at the core of human cognition. Notably, we distinguish between “abstraction,” i.e., the process leading to the formation of categories and order them hierarchically, and the related but independent notion of “abstractness,” a characteristic of abstract concepts (Borghi, 2022). Scholars have a growing consensus that abstract concepts evoke sensorimotor experiences, although they are more detached from them than concrete concepts. Specifically, feature production tasks showed that, with abstract concepts, participants produce sensorimotor and interoceptive properties (Harpaintner et al., 2018; Banks et al., 2021). Rating tasks showed that participants judged that sensorimotor features and effectors such as the mouth and hand are involved in abstract concepts (Ghio et al., 2013; Villani et al., 2019). Finally, brain imaging evidence reveals that sensorimotor brain areas are recruited during abstract concept processing (Sakreida et al., 2013; Kiefer and Harpaintner, 2020). Typically, abstract concepts do not refer to single objects or entities but rather to situations, events, and feelings. Compared to concrete concepts, they evoke more interoceptive, inner bodily experiences and are more variable across individuals, languages, and cultures. Notably, concrete and abstract concepts are not dichotomously opposed; we can distinguish concrete and abstract categories into subkinds. Within concrete categories, the most widely investigated include artifacts, natural objects (Humphreys and Forde, 2001), and food (Rumiati and Foroni, 2016). As to abstract concepts, various sub-kinds exist too, like emotional (Ponari et al., 2018), numerical (Fischer and Shaki, 2018), social (Mellem et al., 2016), and Theory of Mind concepts (Desai et al., 2018; review: Conca et al., 2021). For each of these conceptual kinds, different dimensions—sensorimotor, interoceptive, linguistic, social—might be varyingly relevant in a flexible and contextual-dependent way (Borghi, 2022). For example, emotional concepts rely more on interoceptive experience (Connell et al., 2018; Villani et al., 2021b) and institutional concepts on linguistic and social ones, but expertise modulates the relevance of these dimensions (Villani et al., 2021a).

## Abstract Concepts and Belief

### Belief During Abstract Concepts Acquisition and Use

Why might research on abstract concepts be relevant to literature on beliefs? We contend that it is significant for various reasons. First, because of the mechanism underlying abstract concepts' processing, which entails forming relevant beliefs on the conceptual content and the status of our own knowledge. Literature on categorization reveals that children and adults form relevant beliefs on categories, fostered by language generics (e.g., “sharks attack swimmers”: Gelman et al., 2010; Gelman and Roberts, 2017). We propose that such beliefs are particularly relevant for forming and using abstract concepts. In our view, being more complex than concrete concepts, abstract ones involve a monitoring process (Borghi et al., 2019; Borghi, 2022); individuals assess the reliability of their knowledge. If we define beliefs as meaningful probabilistic representation (Seitz et al., 2018), then during conceptual processing, we would form a belief, which might not be explicit, about the conceptual content and the level and status of our knowledge of a given domain. While the outcome of this monitoring process is typically successful with concrete concepts, this is often not the case with abstract ones. We continuously seek evidence to confirm (and eventually confute) our beliefs, the character of which is dynamic and changeable. Because abstract concepts are complex and indeterminate in meaning, the beliefs concerning their content might be easily disconfirmed and require updating. Consistently, we typically feel more uncertain of our knowledge and understanding of abstract than concrete concepts. As recent evidence suggests, this feeling of uncertainty and low confidence can sometimes become explicit. A rating study demonstrated that people are less confident with abstract than concrete concepts (Mazzuca et al., 2022). Curiously, participants were not only less confident in their own knowledge, but they were also less trustful about experts' knowledge of the same domains.

We posit that a second mechanism showcasing the relationship between abstract concepts and beliefs enters into play when the monitoring process fails. We have called social metacognition (Borghi et al., 2018) the process occurring when we fail to find responses in ourselves and resort to others (Shea, 2018). Consistent evidence shows that participants performing an image-word guessing task tend to ask more for advice from others with abstract compared to concrete words; when performing a subsequent joint action task they show more inter-individual synchrony (Fini et al., 2021a). Furthermore, much evidence showed higher activation of the mouth motor system during abstract than concrete concepts acquisition and processing (review: Mazzuca et al., 2021; see also Ghio et al., 2013; Dreyer and Pulvermüller, 2018; Fini et al., 2021b; Reggin et al., 2021). One possible explanation for this activation is that we might prepare to ask others for information. When uncertain about word meaning, we might appeal to others.

We propose that this process might occur explicitly because we think that others might contribute to our knowledge, or it might be simply an automatic tendency. We might resort to others for various reasons. First, we might need them as experts who explain a concept or help us grasp it more in-depth. Forming beliefs about the authority and expertise of various people might help us select our possible interlocutor. Second, we might appeal to others because we struggle to understand their understanding of a concept or beliefs about its conceptual content. To illustrate, we might attempt to understand our interlocutor's notion of “freedom” to maximize the conversation's quality and mutual understanding. Notably, in some cases, people might violate these maxims and deliberately misinterpret others, especially when their ideological positions differ from their interlocutor's. Finally, we might rely on others because abstract concepts, having a less determined meaning, are more contestable than concrete concepts (Mazzuca and Santarelli, 2022). So, we might feel the need to negotiate their meaning with others. For example, imagine two scientists defining the notion of “belief.” In this case, the two scientists will compare their beliefs on the notion to reach a consensus. Importantly, people might adopt different strategies to evaluate a source as reliable. For example, many people might consider a source reliable if the source agrees with them and unreliable in the opposite case. However, developmental literature on testimony reveals that, even if children tend to favor informants of their ingroup, they revoke their trust in familiar teachers if aware that they receive inaccurate information and select informants who do not flaunt confidence (Kominsky et al., 2016). Reliance on others is clearly more complex when the novel information pertains to issues that are the object of different ideologies.

Similarly, recent findings show that people are more uncertain about abstract concepts and tend to revolve more toward others. For instance, in a recent study, participants had to imagine a dialogue with an acknowledgment; they were prompted by a sentence including different kinds of abstract and concrete concepts (e.g., “I thought about destiny” vs. “I saw a lion”) and had to write a possible response. With abstract concepts, and particularly with the more abstract among them, i.e., philosophical-religious concepts, participants asked more “why” and “how” questions—as opposed to “what” and “where” questions. In addition, they were keener to relaunch the conversation instead of closing it (e.g., “Explain me better”) and used more words signaling uncertainty (e.g., “mmm”) (Villani et al., 2022). This might be because the indeterminate character of the former might leave more space for the conversation to grow and for the exposition and possible alignment of idiosyncratic beliefs to emerge. Finally, Fini et al. (in preparation) found that during a virtual conversation, the psychological closeness between the interlocutors increased as a function of the importance attributed to the other's contribution to the dialogue. The other's contribution was especially relevant when the conversational topic was abstract. Conversing about an abstract topic might foster the creation of common beliefs; these processes of intellectual agreement might boost the overlapping between self-other representations.

To sum up, the study of abstract concepts might be significant for research on belief because during the use of abstract more than concrete concepts, we form a belief in the reliability and solidity of our knowledge, and because we seek to understand more the beliefs of others in given domains (see Figure 1—An example of the mechanisms described: the abstract concept “Self-determination”).

**Figure 1:**
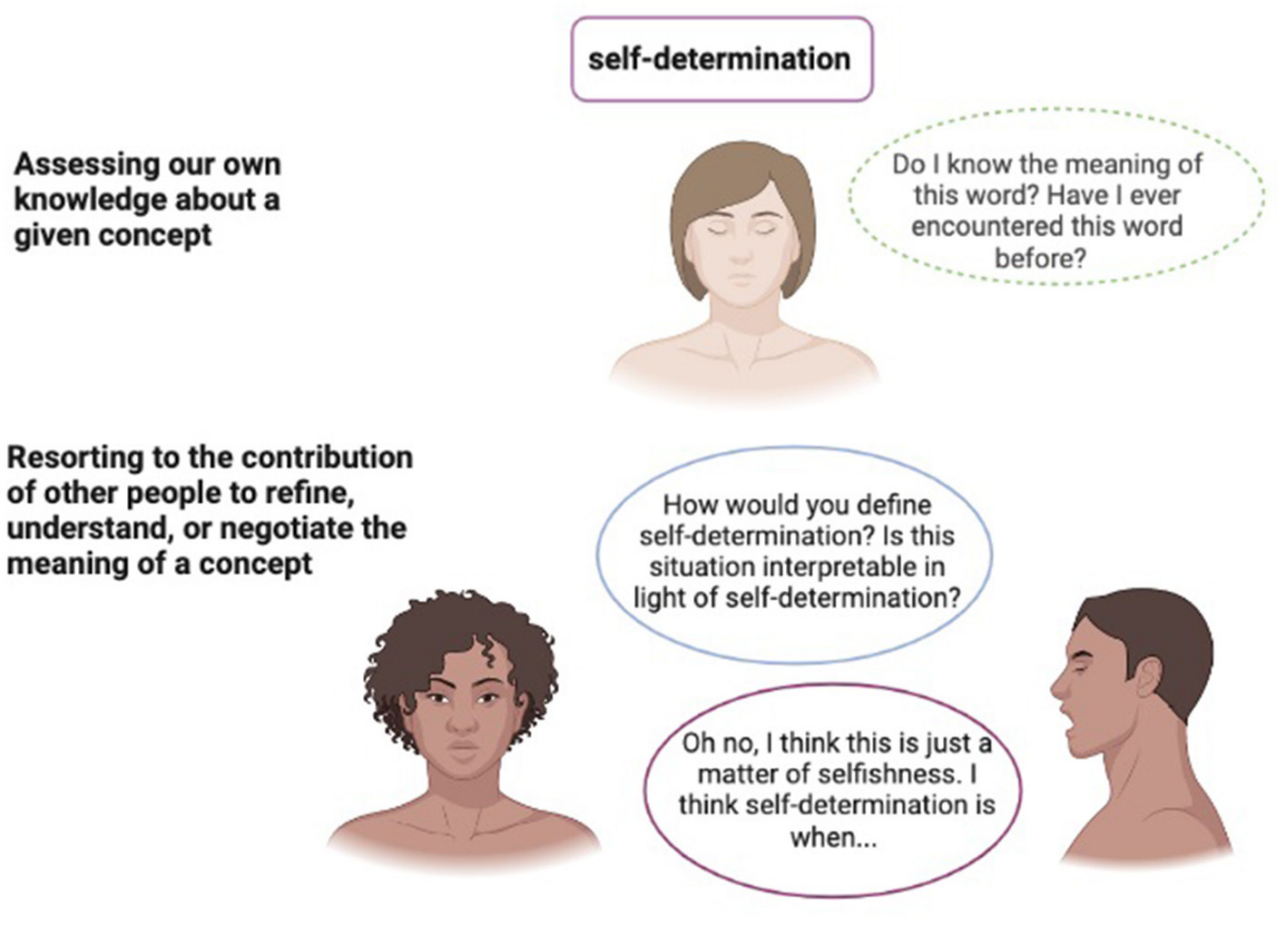
The process of social metacognition.

**The abstract concept of belief:** There is another, probably more straightforward way studies on abstract concepts are relevant to the literature on beliefs. The notion of belief is inherently abstract, and those that are commonly called conceptual beliefs (Seitz and Angel, 2020) represent a sub-kind of abstract concepts. Research on abstract concepts can contribute to understanding how laypeople conceptualize the notion of belief. Notably, however, what people think about beliefs and how they actually use them are not necessarily strictly interrelated. In a study targeting 425 Italian abstract concepts, Villani et al. (2019) asked participants to evaluate them according to various dimensions, including imageability, contextual availability, activation of the five senses, interoception, metacognition, and sociality. A Principal Component Analysis revealed these concepts were characterized by three main dimensions (i.e., sensorimotor aspects, inner grounding, and abstractness~concreteness), and four distinct clusters were identified. Among these, one cluster is predominantly composed of the more abstract among abstract concepts, which are more detached from sensorimotor and inner bodily experiences. We called these Philosophical-Spiritual abstract concepts. Notably, this cluster encompasses several mental states concepts, including “belief,” together with concepts like “religion,” and “faith.” Further information on how belief is conceptualized comes from research on the neural underpinnings of concepts; for example, evidence shows that the brain representation of Theory of Mind concepts, like “belief,” partially overlaps with the regions engaged by moral and emotional concepts (Desai et al., 2018).

## Conclusion

To conclude, in this paper, we identified two main reasons why investigating abstract concepts can be useful for research on beliefs. First, we suggested that the mechanisms underlying abstract concepts processing and use involve multiple forms of beliefs. This is evident in the assessment of the solidity of our knowledge and the knowledge of our interlocutors. Second, we proposed that studies on abstract concepts can provide insights into how we represent the notion of belief in our brain and mind. We hope that future research will produce exciting new findings in this novel area.

## Author Contributions

AB drafted the paper. CM and CF revised and integrated it. All the authors conceived the article. All authors contributed to the article and approved the submitted version.

## Funding

This paper is funded by Dr. Rüdiger Seitz, via the Volkswagen Foundation, Siemens Healthineers, and the Betz Foundation. All authors are funded by the H2020 project TRAINCREASE from social interaction to abstract concepts and words, grant proposal n. 952324. AB was funded also by the Sapienza Excellent Grant-Concepts in interaction with others and with ourselves: Abstractness in social interaction, metacognition and mind wandering, proposal n. RG12117A5D1EB0B3.

## Conflict of Interest

The authors declare that the research was conducted in the absence of any commercial or financial relationships that could be construed as a potential conflict of interest.

## Publisher's Note

All claims expressed in this article are solely those of the authors and do not necessarily represent those of their affiliated organizations, or those of the publisher, the editors and the reviewers. Any product that may be evaluated in this article, or claim that may be made by its manufacturer, is not guaranteed or endorsed by the publisher.
